# Dispersive biofilm from membrane bioreactor strains: effects of diffusible signal factor addition and characterization by dispersion index

**DOI:** 10.3389/fmicb.2023.1211761

**Published:** 2023-07-25

**Authors:** Wonjung Song, Junhee Ryu, Jaehyun Jung, Youngjae Yu, Suyoung Choi, Jihyang Kweon

**Affiliations:** ^1^The Academy of Applied Science and Technology, Konkuk University, Seoul, Republic of Korea; ^2^Department of Civil and Environmental Engineering, Konkuk University, Seoul, Republic of Korea; ^3^HANSU Technical Service Ltd, Sungnam-si, Gyeonggi-do, Republic of Korea; ^4^Department of Chemical and Environmental Engineering, University of Arizona, Tucson, AZ, United States; ^5^Department of Environmental Engineering Konkuk University, Seoul, Republic of Korea

**Keywords:** membrane bioreactor, *cis*-2-Decenoic acid, diffusible signal factor (DSF), quorum sensing, biofilm, extracellular polymeric substances (EPS)

## Abstract

**Introduction:**

Biofilm occurs ubiquitously in water system. Excessive biofilm formation deteriorates severely system performance in several water and wastewater treatment processes. Quorum sensing systems were controlled in this study with a signal compound *cis*-2-Decenoic acid (CDA) to regulate various functions of microbial communities, including motility, enzyme production, and extracellular polymeric substance (EPS) production in biofilm.

**Methods:**

The addition of CDA to six strains extracted from membrane bioreactor sludge and the *Pseudomonas aeruginosa* PAO1 strain was examined for modulating biofilm development by regulating DSF expression.

**Results and discussion:**

As the CDA doses increased, optical density of the biofilm dispersion assay increased, and the decrease in EPS of the biofilm was obvious on membrane surfaces. The three-dimensional visual images and quantitative analyses of biofilm formation with CDA proved thinner, less massive, and more dispersive than those without; to evaluate its dispersive intensity, a dispersion index was proposed. This could compare the dispersive effects of CDA dosing to other biofilms or efficiencies of biofouling control practices such as backwashing or new cleaning methods.

## 1. Introduction

Membrane separation processes including membrane bioreactors (MBRs) are widely applied in wastewater treatment, water reuse, and reclamation. The biggest weakness of the membrane process is that, as the filtration process proceeds, fouling gradually arises on the membrane surfaces. Biofouling has been recognized as the most problematic fouling to control and occurs when microorganisms accumulate and cultivate on the membrane surface (Nguyen et al., 2012; Gkotsis et al., 2014; Tijing et al., 2015). Biofilm formation causes severe performance loss to the membrane system because it yields a shutdown of the process and membrane replacement when the output water quality cannot be sustained through costly cleaning and extensive maintenance (Flemming, 2011; Nguyen et al., 2012; Siebdrath et al., 2019; Maletskyi, 2020; Obotey Ezugbe and Rathilal, 2020). These problems occur more frequently in the MBR process for treating wastewater which contained hazardous substances. Hazardous substances reduce the process performance by weakening microbial activity, and promote biofouling by accelerating or increasing biofilm formation on membrane surfaces. EPS can be increased by humic acid present in the bulk (Ryu et al., 2021), and the increased hazardous substances load in influent develop a denser and thicker biofilm on the membrane surface and have a significant effect on the increase in mean thickness and the viability of the biofilm (Zheng et al., 2022).

Many methods have been developed to control biofilms on membrane surfaces. Extensive pretreatment has been applied to reduce biofouling, mainly by decreasing particulate and organic matter concentrations in the system (Nguyen et al., 2012). Enhancement of the backwashing and *in-situ* cleaning processes was also sought to detach or remove foulants from membrane surfaces. However, technologies to regulate microbial behaviors such as adhesion to the surface, secretion of extracellular polymeric substances, and built-up biofilm structures, for improving biofouling reduction, are still in their early stages. Recently, studies have reported the use of intermicrobial signaling materials as biofouling control strategies (Shahid et al., 2020; Wan et al., 2022; Song et al., 2023). The quorum sensing (QS) system is a communication system involved in the generation of *in vitro* metabolites of microbial cells and communities. Diffusible signal factors (DSF) are a signaling family used in QS systems (Papenfort and Bassler, 2016; Dow, 2017). The DSF family of signal substances are fatty acid-assisted compounds, such as *cis*-2-Decenoic acids (CDA), and regulate various functions of microbial communities, such as motility, enzyme production, EPS production, stress response, and antibacterial resistance (Marques et al., 2015). The regulation of DSF expression can be therefore used to control behavioral patterns of biofilm formation by microbial cells.

A second-messenger molecule, cyclic dimeric guanosine monophosphate (c-di-GMP), is the key factor in the transition from a motile planktonic lifestyle to fixed biofilm formation. Increasing intracellular c-di-GMP levels accelerates exopolysaccharide synthesis, which enhances the induction of biofilm formation and surface aggregation. Binding of DSF to the signal conversion component stimulates the c-di-GMP phosphodiesterase activity of the protein, and biofilm dispersion is induced because the intercellular c-di-GMP level is consequently lowered (Schmid et al., 2012; Suppiger et al., 2016). Biofilm dispersion occurs at the final stage of biofilm development, and microbial cells are released into the bulk liquid. The induction of dispersion is an important control measure for persistent biofilms in wastewater treatment, because dispersion is a mechanism by which bacteria escape overcrowding or unfavorable conditions, allowing fixed cells to migrate to bulk liquids (Davies and Marques, 2009).

Biofilms are composed of a matrix of microorganisms attached to a solid surface and EPS around the outer surface of the organisms (Costa et al., 2018; Karygianni et al., 2020). The EPS produced by microorganisms, regardless of the growth environment, performs several functions, such as stabilizing the biofilm structure and forming a protective barrier against stressed environments (Laspidou and Rittmann, 2002; Joo and Aggarwal, 2018). Loosely and tightly bound EPS produce a substantial filtration resistance, which is an important reason for membrane fouling in MBRs (Teng et al., 2020). The major constituents of EPS are polysaccharides and proteins. Polysaccharides determine the physical properties of biofilms because they contribute to their adhesion, cohesion, scaffolding, stability, intercellular bonding, and antimicrobial protection (Karygianni et al., 2014; Turnbull et al., 2016; Ogran et al., 2019; Reichhardt and Parsek, 2019). Proteins are another important component of the matrix and contribute to the structural stability of biofilms by promoting cell adhesion and aggregation between bacterial cells, leading to the development of designed cell clusters, that is, microcolonies (Drescher et al., 2016; Bowen et al., 2018; Duanis-Assaf et al., 2018). DSF, such as CDA, are responsible for inducing dispersion in microbial biofilms; therefore, increasing CDA concentration in the bulk yield variations in EPS compositions and structures of microbial biofilms (Marques et al., 2014; Sepehr et al., 2014).

The degree of dispersion was examined by measuring the optical density of released cells, microscopic observation of disaggregation of microcolonies, and quantification of dye colorization in a microtiter dish assay (Sepehr et al., 2014; Rahmani-Badi et al., 2015). In addition, three-dimensional image analysis using confocal laser scanning microscopy has been utilized in numerous studies as it provides a visualization of the structure of biofilm and quantitative values of biofilm structure factors such as total biomass, surface-to-biovolume ratio (SBR), mean thickness, and roughness using a specific software of the CLSM image analysis program. However, the degree of dispersion varies greatly with the experimental conditions, making it difficult to evaluate the effects of control technologies for biofouling reduction and determination of operational conditions in water processes.

The integrated index approach has been used in several applications, including risk assessment for drought and climate change, because this approach needs to integrate a wide range of relevant features, especially owing to complicated factors such as physical, social, and environmental elements (Sullivan et al., 2006; Chang et al., 2016). The climate vulnerability index (CVI) was developed using six potential variables: resource, access, capacity, use, environment, and geospatial components. One of the component, for instance, environment included livestock and human population density, loss of habitats, and flood frequency; which provided insights into the vulnerability situation in many different cases. This integrated index approach seemed proper to assess the degree of biofilm dispersion (that is dispersion index, DI) since various factors such as roughness, water channel structure and biomass were related to determine dispersive properties of biofilm.

In this study, the control of microbial dispersion by DSF was examined to determine the possibility of biofouling inhibition technology in the MBR operation of wastewater treatment processes. Previous studies have examined the dispersion induction of strains in dental plaque and biofilms from a variety of single strains, such as *P. aeruginosa* and *P. mirabilis*, by CDA addition (Davies and Marques, 2009; Rahmani-Badi et al., 2015). However, few attempts have been made to apply the DSF system to control biofilms in membrane bioreactors for wastewater treatment, where heterogeneous strains work together to degrade organic matter in water. This study aimed to develop a biofouling abatement technology by interfering with the expression of the QS system by adding CDA, which controls the c-di-GMP level in the DSF system. The effects of CDA addition on strains extracted from MBR sludge, in addition to *Pseudomonas aeruginosa* PAO1, were investigated, focusing on the variation in EPS composition. The degree of dispersion was examined using the optical density of released cells, three-dimensional image CLSM, and changes in polysaccharides and proteins of EPS. In addition, the DI was introduced as a useful tool for comparing numerous inhibitory technologies for dispersion effects.

## 2. Materials and methods

### 2.1. Bacterial strains and growth conditions

*Pseudomonas aeruginosa* PAO1 was selected as the single pure culture for biofilm formation. A mixed culture of six strains was used to extend the understanding of the effects of CDA addition to heterogeneous microbial cultures. Six strains were extracted from the MBR sludge: *Pseudomonas aeruginosa* PAO1, *Pseudomonas aeruginosa* PA14, *Pseudomonas aeruginosa* 15422, *Aeromonas hydrophila* 11163, *Escherichia coli*, and *Streptococcus* sp. Some strains were also identified in a previous study from this laboratory. Lade et al. (2014) reported that 12 isolates, including *Aeromonas, Enterobacter*, *Serratia, Leclercia*, *Pseudomonas*, *Klebsiella*, *Raoultella*, and *Citrobacter*, were recognized by a nucleotide BLAST analysis of the 16SrRNA gene sequence from QS signal-producing bacterial isolates in a domestic wastewater treatment plant. The isolated strains were stored below −65°C. Before the experiment, each strain was inoculated into 2.5% LB broth (Difco BD, Franklin Lakes, NJ, United States) and used after 24 h incubation at 28–30°C.

### 2.2. Antibacterial activity of CDA

The minimum inhibitory concentration test was conducted to investigate the effect of CDA on microbial growth. The CDA (≥95.0% HPLC grade) was purchased from Sigma-Aldrich (St. Louis, MO, USA). CDA doses ranged from 0 to 1,000 nM. The maximum dose was chosen based on the results of a study by Jennings et al. (2012), in which a concentration higher than one μM exhibited microbial growth. In addition, the fact that the CDA concentration found in the supernatant of the inoculated PAO1 culture was at nanomolar concentrations was considered (Davies and Marques, 2009). The MIC test procedure is briefly explained as follows: A growth culture of *P. aeruginosa* (incubated for 24 h at 37°C) was prepared in LB broth. Ethanol (10%) was used as a carrier for CDA samples. A CDA solution of 1 mg/mL was diluted to different concentrations of 10, 50, 100, 200, 500, and 1,000 nM in Mueller-Hinton broth. Each solution (1800 μL) was placed in a 24-well culture plate, and 200 μL of the bacterial suspension was injected. After 24 h of incubation at 37°C, bacterial growth was evaluated by adding 200 μL of the colorimetric indicator of 2, 3, 5-triphenyltetrazolium chloride (TTC; Sigma-Aldrich, St. Louis, MO, USA) at a concentration of 5 mg/mL in each well. Thereafter, the plates were incubated again for 1 h at 37°C, and the intensity of coloration was observed.

### 2.3. Biofilm dispersion assay

The biofilm was grown on a petri dish and the optical density (OD) of the suspension in the petri dish was measured to evaluate biofilm dispersion, since dispersed cells were released into the bulk liquid. The biofilm was grown on a Petri dish by changing the medium every 24 h, which is a semi-batch culture method. After overnight incubation, the culture was diluted with 15 mL of the growth medium, inoculated into a sterile Petri dish, and incubated at 30°C on a shaker at a mixing speed of 30 rpm. The medium was changed every 24 h for three days. On the third day after the last change in the medium, the cells were incubated for approximately 1 h and then replaced with fresh medium containing CDA. Microbial cells were incubated for an additional hour and the medium containing the dispersed cells was separated by sonication for 30 s. CDA concentrations of 100, 200, and 300 nM were used. The cell density in the suspension was determined by measuring OD600 using a UV/VIS spectrophotometer (DR6000, Hach Co., United States). The biofilm dispersion assay was repeated three times for each concentration. The carrier control containing medium plus 10% ethanol was also evaluated in parallel.

### 2.4. Operation of Center for Disease Control (CDC) biofilm reactor

The effect of CDA concentration on biofilm formation was investigated using a Center for Disease Control (CDC) biofilm reactor (BioSurface Technologies Corp., Bozeman, MT, United States). The CDC reactors, a reservoir for medium, polypropylene coupon holders, magnetic bars, and tubes were autoclaved at 121°C for 20 min prior to conducting the experiments for biofilm formation. A commercial microfiltration flat membrane (Merck Millipore, Darmstadt, Germany) composed of polyvinylidene fluoride (PVDF) was purchased. The membrane with a pore size of 0.22 μm was cut into 1.5 cm x 1.5 cm pieces and sterilized using 40% ethanol, followed by UV light exposure for 1 h. The membrane specimen was then fixed on one side of the coupon holder using a double-sided cellophane tape. Two specimens were attached to a coupon rod, and eight rods were attached to the CDC reactor. The CDC reactor was operated at 150 rpm for 24 h. All processes were conducted on a clean bench.

Microbial strains were prepared as follows. The PAO1 culture or the multi-strains were incubated in 2.5% LB broth and filled in the CDC reactors at a concentration of 10^6^–10^7^ CFU/mL. Then, the CDA concentration was determined as 100 nM, 200 nM, and 300 nM by adding 6.8 μL, 13.6 μL, and 20.4 μL of l mg/mL CDA solution (w/10% ethanol), respectively. The total volume of the mixture was 400 mL. A CDC reactor, as a blank control, was also operated under the same conditions without adding CDA. After 24 h of operation, the membranes were removed for EPS and optical imaging analyses.

### 2.5. Extraction and measurement of extracellular polymeric substance (EPS)

EPS formed on the membrane surface was extracted using a thermal method. The membrane was carefully removed from the CDC rod and the residue was washed with 20 mL of 0.9% NaCl solution. After washing, the membrane was transferred to a conical tube containing 15 mL of 0.9% NaCl solution, vortexed for 5 min, and sonicated for 60 min (B5510; Branson Ultrasonics, United States). The membrane was then removed from the solution and centrifuged (5,000 × g, 20 min, 4°C) to extract the biofilm. After centrifugation, the supernatant was removed to obtain the biofilm fraction. After adding the same liquid amount of 0.9% NaCl solution, it was heated in a drying oven at 100°C for 60 min and then cooled to room temperature. After centrifugation (5,000 × g, 20 min, 4°C), the supernatant was removed and filtered with a 0.45 μm syringe filter, and the filtrate was collected. EPS is defined as the sum of proteins and polysaccharides.

The proteins were quantified using the Bradford method. Briefly, a standard curve was obtained using several concentrations of bovine serum albumin (BSA). Standard solutions were prepared by diluting a stock solution with 2 mg/mL BSA in the range 0–10 μg/mL, and the absorbance was analyzed for protein quantification. Each sample and 1 mL of protein dye were placed in a microcuvette and allowed to stand for 10 min. The absorbance was measured at 595 nm. The protein contents of the samples were calculated from the absorbance data of the standard solutions. The protein contents of the samples were calculated from the absorbance data of the standard solutions. The regression curve was obtained as $y=0.0602x+0.0031,r^{2}=0.9971$ . A Protein Assay Kit (BR500, Bio-Rad, United States) was used to analyze the BSA solutions and dye. A Genesys 10 UV/vis spectrophotometer (Thermo Scientific, USA) was used for absorbance measurements. The amount of polysaccharides was measured using a TOC analyzer (SIEVERS 5310C, GE, Australia). The amount of EPS was then divided by the area of the membrane specimens.

### 2.6. Bacterial strains and growth conditions

Confocal laser scanning microscopy (CLSM) analysis was performed to observe biofilms on membrane surfaces. The experimental methods have been described by Lade et al. (2017). Briefly, the detached membranes were dyed for 30 min with 200 μL SYTO 9 (Molecular Probes, Eugene, OR, USA) and wrapped in aluminum foil to block light. Excess stain was carefully washed with deionized sterile water, and the membranes were mounted on glass slides (covered with a coverslip). Microscopic observation and image acquisition were performed on stained membranes using confocal laser scanning microscopy (LSM 710, ZEISS, Germany). The membrane surface was observed at 20 × magnification using CLSM. The observed area was 1,024 × 1,024 μm^2^, with a resolution of 1,024 × 1,024 pixels. The biofilm structure was quantified from the confocal stack using COMSTAT image analysis software. In this study, the biofilm differences generated under each condition were determined using the four COMSTAT parameters. These parameters were the total biomass, surface-to-biovolume ratio (SBR), mean thickness, and roughness coefficient.

The roughness coefficient (R_a_) indicates variability in the measured biofilm thickness (Murga et al., 1995). The formula for calculating is as follows:


$$R_{a}^{*}=\frac{1}{N}\overset{N}{\underset{i=1}{\sum }}\frac{\left|L_{fi}-\bar{L_{f}}\right|}{\bar{L_{f}}}$$

where 𝐿_𝑓𝑖_ is the i-th measured individual thickness, 𝐿_𝑓_ is the average thickness, and N is the number of thickness measurements.

### 2.7. Determination of dispersion index (DI)

An integrated index approach has been adapted to assess the degree of biofilm dispersion in this study. The integrated index was developed to describe a wide range of relevant features on establishment of dispersive biofilm. The experimental factors, including cell density, EPS, total biomass, SBR, thickness, and roughness, were examined to represent biofilm dispersion, and the relevant factors were used to calculate the DI of the biofilm.

The correlation of several factors, such as structural properties, was considered for selecting appropriate variables for inclusion in the DI framework. The variables for DI included OD values and two biofilm structural factors from COMSTAT analyses, that is, SBR and roughness. The value of DI was calculated as a weighted average of all components, as shown below, for which the equation in Sullivan et al. (2006) was followed.


$$DI=\frac{ro*O.D+rs*SBR+rr*Roughness}{ro+rs+rr}$$

where the weight given for each component is determined by a factor r representing the relevance of the component. Factor r (i.e., ro, rs and rr) was obtained by fitting the DI and EPS concentrations to obtain the greatest R-squared value, which represents the strength of the correlation between the independent and dependent variables. The R-squared value was 0.9326, which indicated that DI was well correlated with the EPS concentration.

### 2.8. Statistical analysis

Biofilm dispersion was statistically analyzed using Microsoft Excel software (Microsoft, Redmond, WA, United States). The data shown represents the mean values obtained from three independent experiments, with error bars indicating the corresponding standard deviations. Biofilm dispersion induced by the addition of CDA was evaluated in terms of the Statistical significance. *p* value <0.05 is considered statistically significant.

## 3. Results and discussion

### 3.1. Effects of CDA addition on dispersal and EPS concentrations in biofilm

**Figure 1:**
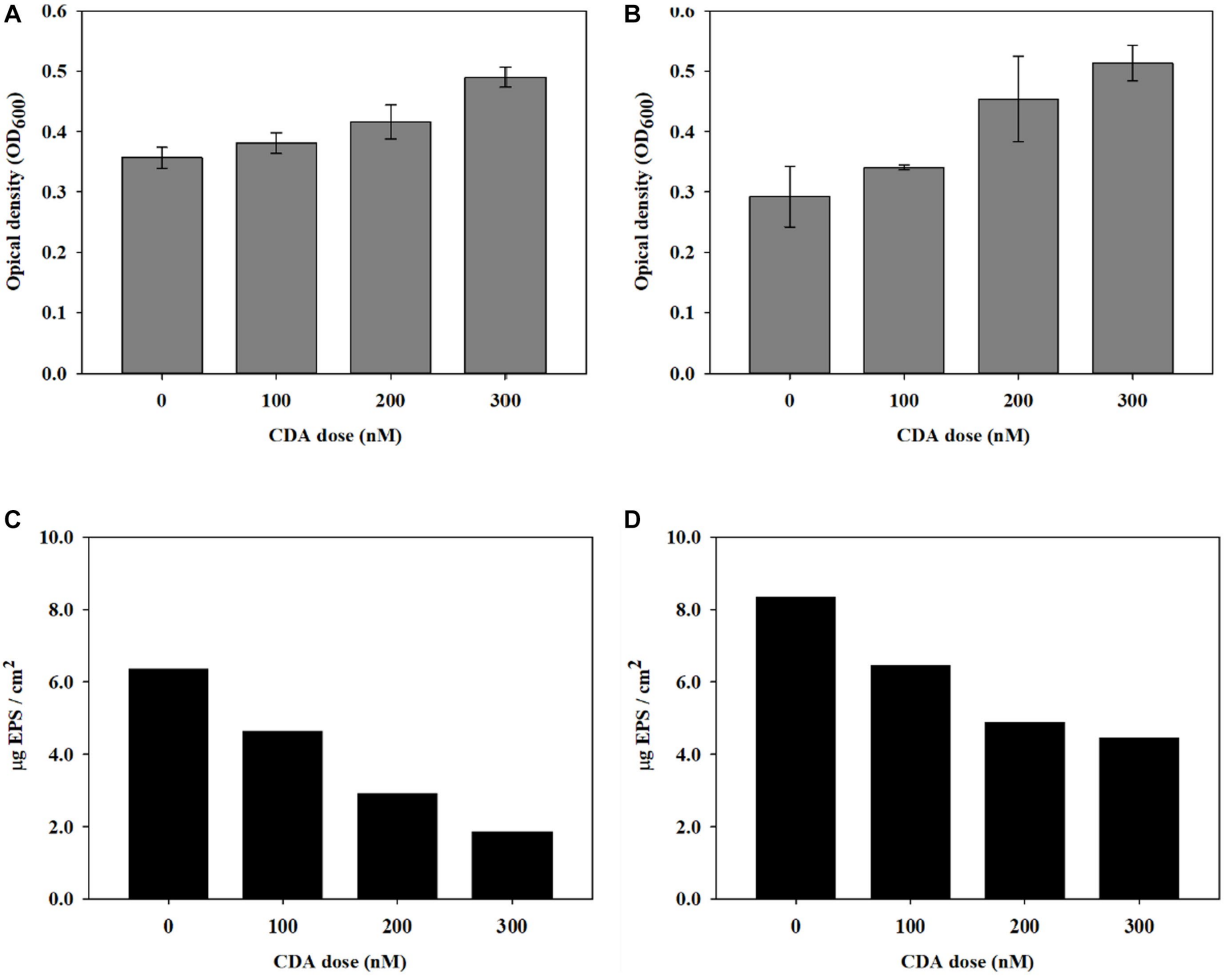
Induction of biofilm dispersal by CDA addition on **(A)** PAO1 and **(B)** multi-strains (*n* = 3) and variations in EPS concentration on biofilms by **(C)** PAO1 and **(D)** multi-strains.

The dispersion was evaluated by measuring OD at 600 nm; the results are shown in Figure 1. The OD values (*n* = 3) of the blank experiment with the PAO1 strain and without the addition were 0.357 ± 0.017, as shown in Figure 1A. Compared with the control experiment, the OD values increased with CDA addition. An OD value of 0.490 ± 0.016 was observed at a CDA concentration of 300 nM. The results indicate the occurrence of an increase in planktonic cell populations, probably owing to the release of microbial cells from the biofilm to the bulk liquid when CDA was added to the PAO1 strain. The OD values of the multi-strain experiments are shown in Figure 1B. Also, OD values (*n* = 3) increased with increasing CDA concentrations. The OD value was 0.292 ± 0.050 in the experiment without CDA, 0.381 ± 0.017 with 100 nM CDA, 0.416 ± 0.028 with 200 nM CDA, and 0.490 ± 0.017 with 300 nM CDA. The variation in the OD values with increasing CDA was relatively large in the multi-strain experiments. The statistical analysis between the application and non-application of CDA revealed significant differences in both the PAO1 and multi-strain experiments, with *p*-values of 0.042 and 0.011, respectively. Davies and Marques (2009) showed the effects of dispersion of different bacterial biofilms, including PAO1, by CDA using a microtiter plate dispersion bioassay with a 4-day cultivation. The results indicated an increase in released cells (evaluated with OD570) with 10 nM CDA dose for the experimented bacteria such that the dispersion efficacy reached 24.6%. The authors suggested further studies on biofilms with multiple species and the impacts of the degradation of extracellular polymers produced by neighboring microorganisms of other species. In contrast to passive detachment, dispersion is an active process, involving a coordinated response to changes in the surrounding environment and requiring the contribution of cell-to-cell signals (Light, 2017). The higher OD values were detected from PAO1 strain. The high OD was due to numerous factors including debris materials and interference of biomolecules. The changes in OD values with CDA doses were greater in the multiple strains, which may indicate that effective dispersion was achieved by CDA addition, in part, because a variety of microbial cells were involved, demonstrating that induction of dispersion might be a useful technology for biofouling reduction in systems with numerous bacterial consortia, such as an MBR for wastewater treatment.

To understand whether the QS compound affected the biofilm composition in addition to dispersal behavior, the EPS concentration of the biofilm was evaluated with CDA addition. CDC reactors were operated to obtain sufficient biofilms for EPS analysis. The variation in EPS concentrations with different CDA concentrations of 100, 200, and 300 nM are presented for PAO1 in Figures 1C,D for multi-strains. The EPS content of the PAO1 biofilm without CDA was 6.37 μg EPS/cm^2^. The EPS of the PAO1 biofilm decreased to 4.64, 2.93, and 1.87 μg EPS/cm^2^ by adding 100 nM, 200 nM, and 300 nM of CDA, respectively. The variations in EPS concentrations of the biofilm formed by multi-stain showed a trend similar to that of PAO1. The total EPS of the biofilm without CDA was 8.34 μg EPS/cm^2^ and decreased to 6.46, 4.90, and 4.47 μg EPS/cm^2^ by adding 100 nM, 200 nM, and 300 nM of CDA, respectively. The EPS declined by 46% at a CDA dose of 300 nM.

Several studies have shown that fatty acid signaling molecules, including CDA, are responsible for inducing biofilm dispersion in a range of gram-negative and gram-positive bacteria (Davies and Marques, 2009; Sepehr et al., 2014; Kumar et al., 2020). They discussed that a variety of saturated and unsaturated fatty acids act as inhibitors of bacterial colonization and biofilm development by affecting the adhering surface, changing cell membrane fluidity, reducing EPS, and modulating QS systems (Kumar et al., 2020). However, the exact mechanisms and important factors that determine the dispersive effects during biofilm development, including the amount and characteristics of EPS, have been poorly established. The measured EPS concentrations of biofilms from PAO1 and multiple strains shown in Figures 1C,D quantitatively revealed that the injection of CDA lowered the amount of EPS in biofilms. Polymeric substances adhere to the membrane surface, block pores in the membrane, and affect cake layer properties, resulting in severe membrane fouling (Teng et al., 2020). Changes in biofilm composition modulate the characteristics of biofilms, such as persistence to shear force and resistance to antibacterial chemicals, which enhance efficiencies in backwash and other cleaning procedures for MBR operation.

The EPS concentration in the biofilm formed by the multi-strain mixture showed relatively high values compared with that of the PAO1 single strain, regardless of the CDA doses, indicating that coordinated patterns of behavior inside biofilms by polymicrobial cells increased interactions and synergic effects between microorganisms; this was related to improvement in tolerance, persistence, and EPS production of the biofilm (DeLeon et al., 2014; Song et al., 2018). The extent of EPS decrease by CDA addition was also lower in the biofilm with multiple strains than that in the PAO1 strain. The formation of biofilms provided a protective barrier against stressed environments; thus, the high concentrations of EPS in the biofilms with multiple strains probably hindered the effects of CDA addition, including dispersion, and vice versa. Understanding the EPS characteristics is necessary for evaluating the effects of CDA addition on biofilm modulation.

### 3.2. Variation in protein and polysaccharide of EPS by CDA addition

EPS is known to be a medium that allows the aggregation of microorganisms and stable proximity of bacteria, thus producing biofilms (Laspidou and Rittmann, 2002; Joo and Aggarwal, 2018). The major components of EPS are polysaccharides and proteins. In general, proteins participate in stabilizing the aggregate structures of biofilms and in the digestion of macromolecules and particular compounds in the surrounding microbial cells (Ryu et al., 2021). Proteins contain high amounts of negatively charged amino acids, and are thus involved in electrostatic bonds with multivalent cations. Laspidou and Rittmann (2002) also indicated that extracellular proteins act as enzymes for the digestion of macromolecules and particulate materials in the microenvironment surrounding biofilms.

**Figure 2:**
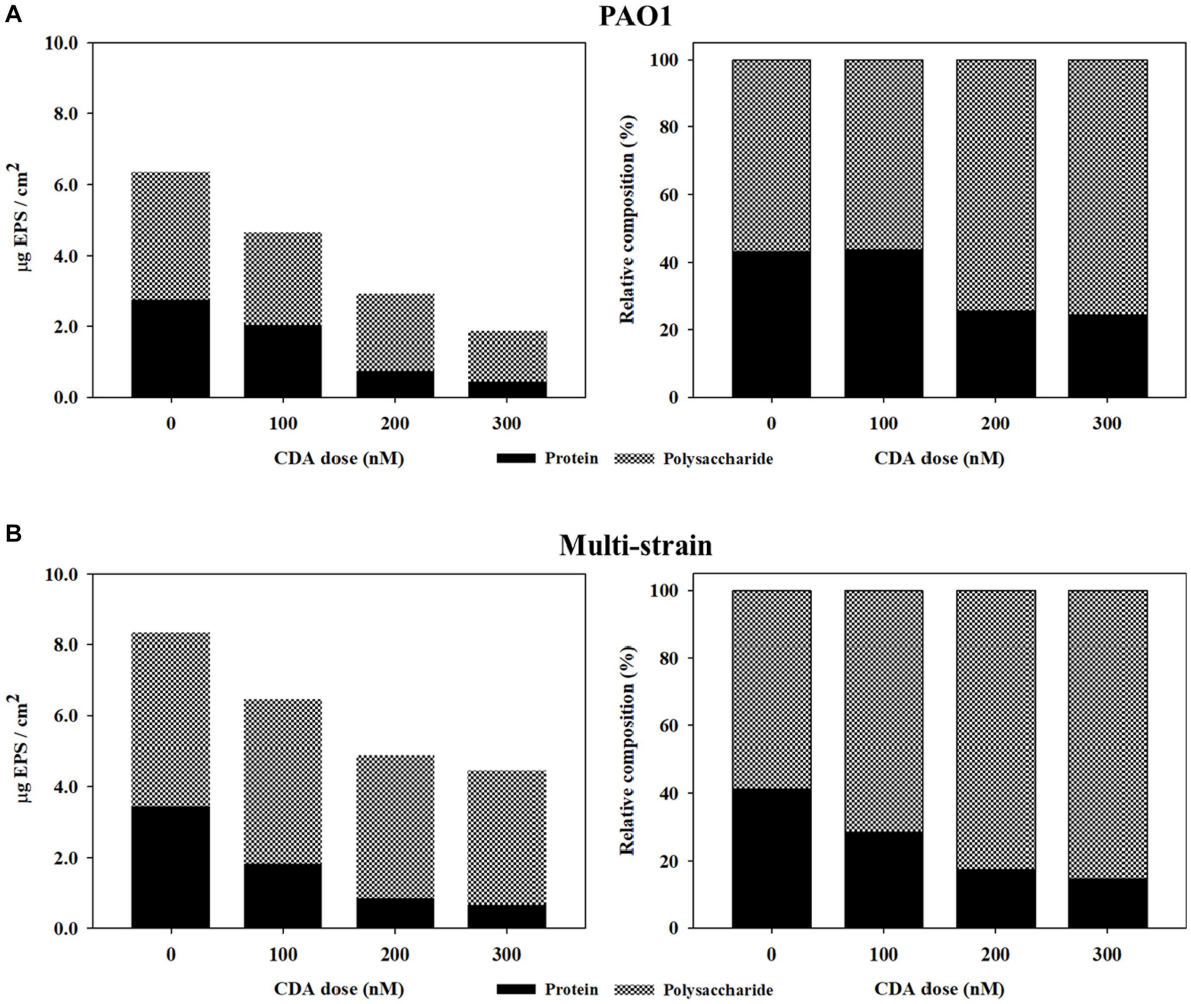
Effects of CDA addition regarding variations in relative compositions of protein and polysaccharide in EPS of biofilms formed by **(A)** PAO1 and **(B)** multi-strains.

The variations in the relative compositions of proteins and polysaccharides in the EPS are summarized in Table 1 and Figure 2. The polysaccharide content in the PAO1 biofilm decreased as the concentration of CDA increased, corresponding to a decrease in the total EPS. The polysaccharide amount of the PAO1 biofilm without CDA was 3.61 μg C/cm^2^ and decreased to 2.61, 2.18, and 1.42 μg C/ cm^2^ by adding 100 nM, 200 nM and 300 nM of CDA, respectively. Protein levels also showed a decreasing pattern with increasing CDA concentrations in the PAO1 biofilm. At a dose of 300 nM, protein was detected at 0.46 μg BSA/ cm^2^ in the PAO1 biofilm and 2.75 μg BSA/cm^2^ in the control sample. As in the PAO1 biofilm, polysaccharide concentrations in the multi-strain biofilm decreased with increasing CDA doses. However, the extent of polysaccharide reduction was rather dampened; as such, the reduction rate of multi-strain biofilm was only 22%, while that of PAO1 biofilm was approximately 61% at a CDA dose of 300 nM. Compared with the polysaccharide, changes in protein content in the multi-strain biofilm were remarkable, as over 80% of reduction was observed at the CDA dose of 300 nM. In PAO1 biofilm, the protein content in the total EPS was 43% at 0 nM CDA dose and declined to 24% at 300 nM. The protein content of the multi-strain biofilm without CDA was 3.44 μg BSA/cm^2^ and decreased to 1.83, 0.85, and 0.65 μg C/cm^2^ when 100 nM, 200 nM and 300 nM of CDA were added, respectively. In multi-strain biofilm, the relative content of protein in the total EPS was 41% at CDA dose of 0 nM and decreased to 15% at 300 nM. Compared with the PAO1 biofilm, the reduction in protein in the multi-strain biofilm was considerably more extraordinary.

**Table 1:** Polysaccharide and protein concentrations in the biofilm formed by PAO1 and multi-strains.

CDA doses (nmol/L)		0	100	200	300
PAO1	Polysaccharide (μg C/cm^2^)	3.61	2.61	2.18	1.42
	Protein (μg BSA/cm^2^)	2.75	2.03	0.75	0.46
Multi-strains	Polysaccharide (μg C/cm^2^)	4.90	4.62	4.05	3.81
	Protein (μg BSA/cm^2^)	3.44	1.83	0.85	0.65

Dispersion involves an active and organized response of microorganisms to changes in the surrounding environment, requiring cell-to-cell communication. To escape the protective EPS matrix in biofilms, cells secrete degrading enzymes such as proteases, lipases, and lyases (Light, 2017). Enzymatic degradation may be a major factor when the strong gel types of adhesive, which anchor microbial cells in the biofilm, are dissolved, leading to a rapid loss of biofilm integrity (Laspidou and Rittmann, 2002). Dispersed cells are distinct in terms of protein production from biofilms and planktonic cells (Sauer et al., 2002). The study divided proteins into four general classes (Class I, II, III, IV, and V), depending on differential regulation during the course of biofilm development. Proteins encoded metabolic processes for adhesion and involved in various bio-synthesis reactions and molecular transport for bacterial extracellular solute-binding proteins, adaptation, and protection. However, the protein types differed significantly at each stage of biofilm development. In the final stage of biofilm development, the dispersion stage allows microbial cells to move back into the bulk liquid to gain better access to nutrients and to leave behind a shell-like structure. Ren et al. (2016) investigated the ratio of proteins and polysaccharides in EPS in a moving-bed biofilm reactor. Depending on the operational temperature, the protein ratio was between 40–60%. Ryu et al. (2021) examined EPS concentrations of mixed liquor in an MBR and demonstrated that, regardless of substrate biodegradability, the fraction of polysaccharide in the bulk was greater than 84%. Sauer et al. (2002) also mentioned that the protein properties in the dispersion stage biofilms were closer to the properties of planktonic bacteria than those of biofilms in the maturation stage, in which a single strain, such as PAO1 or *Pseudomonas putida*, was used to build biofilms. Biofilm dispersion can be influenced not only by the degradation of polysaccharide matrix but also by changes in the protein composition within the biofilm structure (Guilhen et al., 2017). The results of our study indicated that the addition of CDA in the MBR process has the potential to regulate the cell-to-cell signals of multi-strains, resulting in changes in the concentrations and characteristics of proteins within the biofilm. This leads to a significant decrease in the protein fraction, potentially increasing biofilm dispersion.

### 3.3. Variations in biofilm structures by CDA addition

**Figure 3:**
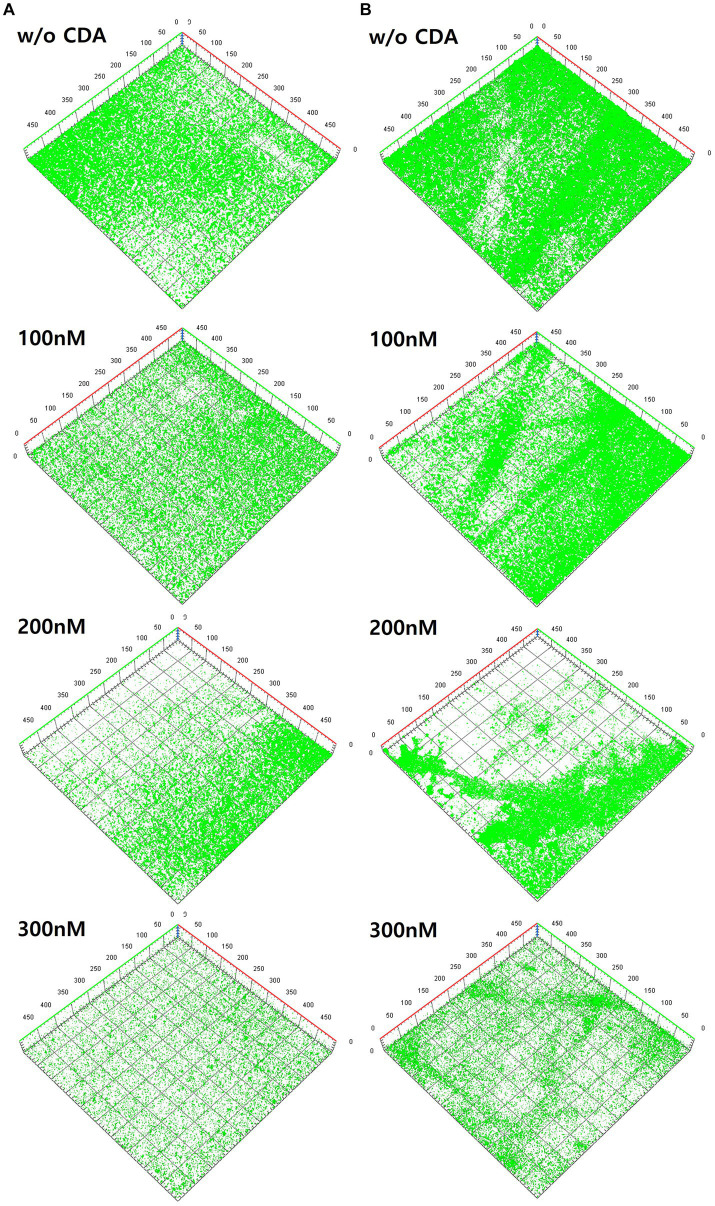
Variations of three-dimensional structures of biofilms by different CDA concentrations on **(A)** PAO1 biofilm and **(B)** multi-strains biofilm.

Numerous studies have visualized clustering patterns or particular shapes of biofilm structures in specific biofilm development stages using CLSM images (Karygianni et al., 2014; Hartmann et al., 2021). CLSM analyses were also conducted in this study to observe the biofilm structures of PAO1 and multiple strains by adding CDA, as shown in Figure 3. Several physical properties, such as roughness and thickness, were obtained from the quantification analysis of the CLSM images, as presented in Table 2.

**Table 2:** Biofilm properties from COMSTAT analyses: effects of CDA on PAO1 and multi strain biofilms.

PAO1	Total biomass (μm^3^ μm^−2^)	Surface to biovolume ratio (μm^2^ μm^−3^)	Mean thickness (μm)	Roughness coefficient
0 nM	12.897 ± 3.661	5.476 ± 0.803	38.989 ± 1.877	0.005 ± 0.005
100 nM	5.435 ± 4.319	8.701 ± 3.134	8.474 ± 6.020	0.374 ± 0.357
200 nM	0.109 ± 0.010	18.174 ± 0.108	1.170 ± 0.063	1.967 ± 0.026
300 nM	0.044 ± 0.021	19.300 ± 0.397	1.003 ± 0.045	1.993 ± 0.007
Multi-strains	Total biomass (μm^3^ μm^−2^)	Surface to biovolume ratio (μm^2^ μm^−3^)	Mean thickness (μm)	Roughness coefficient
0 nM	26.071 ± 2.980	4.237 ± 0.283	43.914 ± 0.679	0.002 ± 0.004
100 nM	17.933 ± 0.961	5.507 ± 0.489	27.977 ± 2.390	0.034 ± 0.048
200 nM	7.619 ± 1.876	6.397 ± 1.163	12.077 ± 1.073	0.051 ± 0.037
300 nM	0.441 ± 0.500	16.373 ± 2.929	11.433 ± 5.535	0.066 ± 0.032

The left-hand side images are from the PAO1 biofilm (Figure 3A), and the right-hand side images are from the multi-strain biofilm (Figure 3B). The image from PAO1 without CDA addition displayed a large amount of green color on the membrane surface, which covered the entire surface of the membrane after 24 h of incubation. Substantial amounts of PAO1 biofilm were clearly visible with a significant green fluorescence intensity in the CLSM image of the membrane. As shown in Figure 3A, the intensity of the green fluorescence of the PAO1 biofilm decreased gradually with increasing CDA concentration. The decrease in green color intensity with CDA doses indicate that extensive amounts of cells fled from the biofilm to the bulk liquid. Previous studies have also shown that shell-like structures with hollow centers and walls of chunks of bacteria were displayed during the dispersion stage because bacteria actively moved away from the interior portions of the cell cluster (Sauer et al., 2002; Marques et al., 2015).

The CLSM images for multi-strain biofilms showed a pattern similar to that of the PAO1 biofilm, such that the greatest intensity was observed in the biofilm without CDA dose and diminished with increasing CDA doses (Figure 3B). In addition, the multi-strain biofilm showed a relatively stronger intensity than the PAO1 biofilm at the same CDA dose, indicating that the multi-strain biofilm was more robust to CDA addition.

CLSM images were used to quantify factors such as total biomass, SBR, mean thickness, and roughness through the image quantitative analysis program, i.e., COMSTAT (An and Parsek, 2007; Reichhardt and Parsek, 2019). The total biomass (μm^3^/μm^2^) was obtained by multiplying the number of biomass pixels in all images by the unit volume of the pixel and dividing by the substratum area. The SBR reflects the fraction of the biofilm that is exposed to nutrient flow. For instance, the ratio supposedly increases with a low nutrient concentration to optimize access to nutrient supply (Heydorn et al., 2000). The values calculated from the COMSTAT program were useful for quantitatively examining the biofilm matrix as well as the amount of adherent biomass (Reichhardt and Parsek, 2019). The biofilm properties obtained from COMSTAT analyses in this study are presented in Table 2. As the CDA doses were increased, total biomass and mean thickness decreased while SBR and roughness coefficient increased for both PAO1 and multi-strain biofilms. The extent of variation at different CDA doses was greater in the PAO1 biofilm than the polymicrobial biofilm from the six strains extracted from the MBR sludge.

In the operating condition without CDA, the total biomass of PAO1 was 12.897 μm^3^/μm^2^, but when CDA was dosed at 300 nM, it decreased considerably to 0.044 μm^3^/μm^2^. The reduction of total biomass by CDA dosing corresponded increasing SBR. A CDA dose of 300 nM on the PAO1 biofilm increased the SBR by approximately 3.5 times (i.e., 19.300 μm^2^/μm^3^) from the SBR without CDA addition (i.e., 5.476 μm^2^/μm^3^). The mean thickness also decreased from 38.989 μm without CDA to 1.003 μm with 300 nM CDA in PAO1 biofilm. The roughness coefficient increased with increasing CDA dose. The addition of CDA to the PAO1 biofilm yielded not only a lower total biomass and thinner biofilm but also more pathways for nutrient flow and greater roughness in biofilm structures compared with those without CDA. The greater values of SBR and roughness with increasing CDA doses might indicate that biofilm development transferred to the dispersion stage; thus, nutrient flow relatively prevailed in the biofilm.

The decreasing or increasing trends of COMSTAT factors in the multi-strain biofilms with increasing CDA doses were the same as those in the PAO1 biofilm. Interestingly, percentage variations in the total biomass, SBR, and roughness of PAO1 biofilm were comparable to those in the multi-strain biofilms, such that the reduction in total biomass at the CDA dose of 300 nM was 99% for PAO1 biofilm and 98% for multi-strain biofilm. The greatest difference was shown with the thickness values: 97.4% in the PAO1 biofilm and 73.9% in the multi-strain biofilm at a dose of 300 nM. The diversity of the mixture of species probably increased the complex patterns of behavior inside the biofilm. The characteristics of polymicrobial biofilms demonstrate that the interactions and synergy of multiple microorganisms affect growth, persistence of biofilm, production of EPS, and biofilm structures (Mastropaolo et al., 2005; Burmølle et al., 2006; DeLeon et al., 2014).

The addition of CDA to PAO1 and multiple strains evidently lowered the green color intensities in biofilms and affected the structural properties of biofilms, indicating an increase in the dispersive characteristics of the biofilm, such as SBR and roughness. Quantifying the degree of biofilm dispersion is necessary for developing strategies for enhancing biofilm dispersion by adding CDA.

### 3.4. Correlation of biofilm dispersion using an integrated index approach

**Figure 4:**
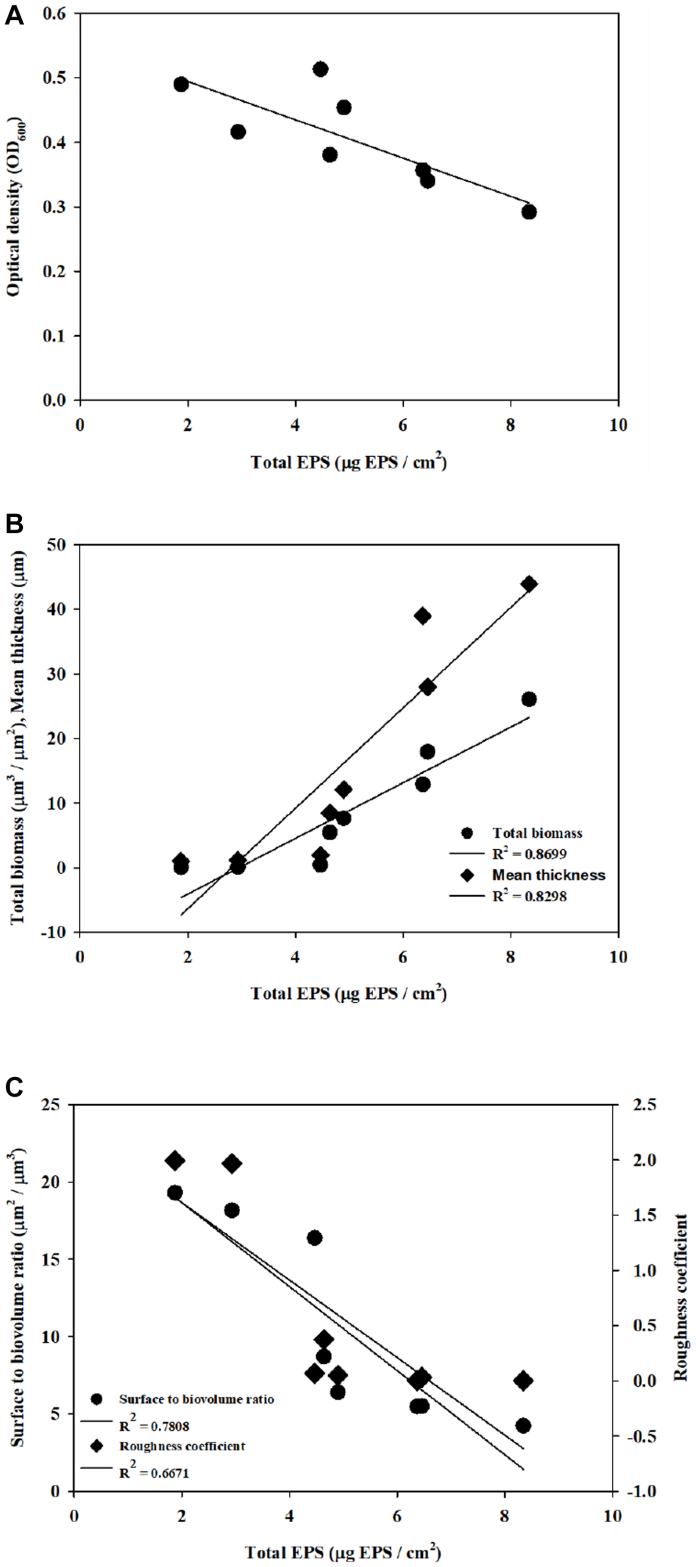
Correlation of structural properties and dispersal with EPS was obtained with **(A)** cell density, **(B)** total biomass and thickness, and **(C)** SBR and roughness.

The application of CDA induced biofilm dispersion (measured by OD values in this study), which resulted in an EPS reduction on the membrane surface (Figure 1). Each factor was correlated with EPS, as shown in Figure 4. Cell dispersal was inversely correlated with EPS concentrations, since dispersive biofilm showed a low concentration of EPS, which was similar to that of bulk liquid. The SBR and roughness also followed the same inverse trend as cell dispersal, whereas total biomass and thickness showed positive correlations with EPS, which was opposite to the trend of OD values.

**Figure 5:**
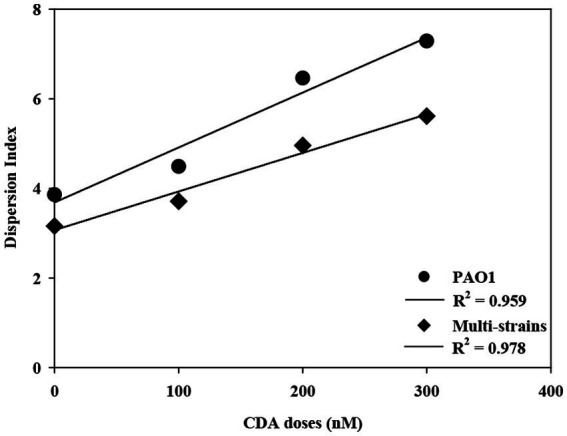
Correlation of DI with increasing CDA doses for PAO1 and multi-strain biofilms.

The degree of biofilm dispersion was proposed to be assessed with the integrated index approach, which was designated as DI for the biofilm. The DI with CDA doses for PAO1 and multi-strain biofilms is shown in Figure 5. The DI increased proportionally with increasing CDA dose, corresponding to the experimental results from the dispersal assay. The DI values also revealed that the PAO1 biofilm was more dispersive than the multi-strain biofilm, which is also consistent with the complexity of the polymicrobial biofilm characteristics. The measurement of EPS, dispersal assays, and COMSTAT analyses are common analytical tools for biofilm studies, and these results were conveniently applied for calculating DI, which provided a quantitative number of biofilm dispersions.

The R-squared (R^2^) of DI values were 0.959 with PAO1 strain and 0.978 with multi-strains. The biofilm formation affected by various factors including a composition of microbial species like single or multiple strains and the surrounding environment like presences of chemicals or signaling compounds. The resulting properties such as roughness and surface to biovolume were also varied significantly corresponding to the complexity of biofilm. The R^2^ was used to evaluate representatives of dispersion index values on effects of CDA addition on the PAO1 and multiple strain biofilms. The correlation could be clearly evaluated by multiple CDA dosage.

The integrated index approach adapted to assess the degree of biofilm dispersion was effective to evaluate the effects of CDA on controlling biofilm structures. The proposed DI would be useful for assessing the degree of dispersion of biofilms; thus, the effects of CDA doses on other biofilms could be compared with DI values, or the effects of biofouling control practices, such as backwashing, could be evaluated with DI values for better cleaning methods.

## 4. Conclusion

The results of this study demonstrated that compared with the polysaccharide fraction, the protein fraction of the total EPS decreased noticeably with increasing CDA doses, implying that adding CDA possibly modified characteristics such as structural stability, cell adhesion, and cell aggregation for dispersive biofilm. The CDA addition yielded biofilms with less total biomass, thinner depth, more pathways for nutrient flow, and rougher structures, compared with those without CDA. Biofilm dispersion was quantified using the DI, and it was useful for comparing biofouling control methods such as backwashing with CDA to determine the most effective cleaning methods. To effectively apply CDA as an advanced biofouling control technology, further research is necessary to evaluate its economic feasibility for practical implementation, explore various application approaches, and conduct ongoing assessments through long-term operation to ensure sustained effectiveness.

## Data availability statement

The original contributions presented in the study are included in the article/supplementary material, further inquiries can be directed to the corresponding author.

## Author contributions

WS: conceptualization, writing – original draft preparation, investigation, and validation. JR and JJ: validation. YY and SC: investigation. JK: conceptualization, writing – review and editing, project administration. All authors contributed to the article and approved the submitted version.

## Funding

This work was supported by a National Research Foundation of Korea (NRF) grant, funded by the Korean government (MSIT) (no. NRF-2021R1A2C2014255).

## Conflict of interest

JJ was employed by HANSU Technical Service Ltd., Republic of Korea.

The remaining authors declare that the research was conducted in the absence of any commercial or financial relationships that could be construed as a potential conflict of interest.

## Publisher’s note

All claims expressed in this article are solely those of the authors and do not necessarily represent those of their affiliated organizations, or those of the publisher, the editors and the reviewers. Any product that may be evaluated in this article, or claim that may be made by its manufacturer, is not guaranteed or endorsed by the publisher.

## Abbreviations

BSA: Bovine serum albumin
CDA: *cis*-2-Decenoic acid
CDC biofilm reactor: Center for Disease Control biofilm reactor
c-di-GMP: cyclic dimeric guanosine monophosphate
CLSM: Confocal laser scanning microscope
CVI: Climate vulnerability index
DI: Dispersion index
DSF: Diffusible signal factors
EPS: Extracellular polymeric substance
MBR: Membrane bioreactor
OD: Optical density
PVDF: Polyvinylidene fluoride
QS: Quorum sensing
SBR: Surface-to-biovolume ratio
